# Transparency in Nigeria's public pharmaceutical sector: perceptions from policy makers

**DOI:** 10.1186/1744-8603-5-14

**Published:** 2009-10-29

**Authors:** Habibat A Garuba, Jillian C Kohler, Anna M Huisman

**Affiliations:** 1 Leslie Dan Faculty of Pharmacy, University of Toronto, 144 College Street, Toronto, Ontario, Canada

## Abstract

**Background:**

Pharmaceuticals are an integral component of health care systems worldwide, thus, regulatory weaknesses in governance of the pharmaceutical system negatively impact health outcomes especially in developing countries [1]. Nigeria is one of a number of countries whose pharmaceutical system has been impacted by corruption and has struggled to curtail the production and trafficking of substandard drugs. In 2001, the National Agency for Food and Drug Administration and Control (NAFDAC) underwent an organizational restructuring resulting in reforms to reduce counterfeit drugs and better regulate pharmaceuticals [2]. Despite these changes, there is still room for improvement. This study assessed the *perceived *level of transparency and potential vulnerability to corruption that exists in four essential areas of Nigeria's pharmaceutical sector: registration, procurement, inspection (divided into inspection of ports and of establishments), and distribution.

**Methods:**

Standardized questionnaires were adapted from the World Health Organization assessment tool and used in semi-structured interviews with key stakeholders in the public and private pharmaceutical system. The responses to the questions were tallied and converted to scores on a numerical scale where lower scores suggested greater vulnerability to corruption and higher scores suggested lower vulnerability.

**Results:**

The overall score for Nigeria's pharmaceutical system was 7.4 out of 10, indicating a system that is marginally vulnerable to corruption. The weakest links were the areas of drug registration and inspection of ports. Analysis of the qualitative results revealed that the perceived level of corruption did not always match the qualitative evidence.

**Conclusion:**

Despite the many reported reforms instituted by NAFDAC, the study findings suggest that facets of the pharmaceutical system in Nigeria remain fairly vulnerable to corruption. The most glaring deficiency seems to be the absence of conflict of interest guidelines which, if present and consistently administered, limit the promulgation of corrupt practices. Other major contributing factors are the inconsistency in documentation of procedures, lack of public availability of such documentation, and inadequacies in monitoring and evaluation. What is most critical from this study is the identification of areas that still remain permeable to corruption and, perhaps, where more appropriate checks and balances are needed from the Nigerian government and the international community.

## Background

Pharmaceuticals are critical for the health and well-being of populations. Their access and consumption can be likened to a double-edged sword: on one hand, they alleviate the manifestation of disease but on the other hand, if they are inappropriately used, or worse, counterfeit or substandard, they may be ineffective and even toxic to the individuals who take them [3-5]. As such, it is necessary for countries to adhere to the highest standards of quality in the manufacture, regulation, and distribution of drugs. Because of the many steps involved in the process of regulating drugs, the pharmaceutical system is particularly vulnerable to unethical and corrupt practices. In many developing countries such as the Federal Republic of Nigeria, these vulnerabilities are capitalized on and adherence to such high regulatory standards is severely impaired by a lack of transparency, weak regulatory control, and the preponderance of corruption in the public pharmaceutical sector [6,7] which negatively impact health outcomes, weaken the nation's economy, and decrease public trust in the government.

Corruption, defined by Transparency International as "the abuse of entrusted power for private gain" [8], was identified as one of the major reasons for the preponderance of counterfeit drugs in Nigeria, in addition to inadequate legislation, ineffective enforcement of existing laws, non-health professionals in the drug business, loose control systems, high cost of drugs, greed, and ignorance [9]. Examples of corrupt practices that facilitated counterfeiting of drugs included extortion of bribes from applicants for drug registration, deliberate over-supply of drug samples for resale, and acceptance of perquisites and material gifts from companies being inspected, to name a few [10].

Counterfeit drugs are defined by the WHO as those that are "deliberately and fraudulently mislabelled with respect to identity and/or source" [6]. Counterfeit drugs fall under the general umbrella of substandard drugs - genuine products which do not meet the quality specifications set for them [10]. Counterfeited drug preparations in Nigeria included those without active ingredients, toxic preparations, expired drugs that were relabelled, drugs issued without complete manufacturer information, and drugs that were unregistered with NAFDAC. Over the past decade and a half, Nigeria struggled to curtail the production and trafficking of counterfeit drugs without adequate infrastructure or the political will to properly enforce legislation and standards.

However, in 2001, under the leadership of Dr. Dora Akunyili as the new Director General of the NAFDAC, the agency underwent dramatic restructuring and reform such as the re-orientation and retraining of NAFDAC staff, establishment of more NAFDAC state offices, refurbishment of drug analysis laboratories, stricter enforcement of drug regulations, public confiscation and destruction of counterfeit drugs, and public awareness campaigns [11]. The aim was to a revitalize NAFDACs mandate to "safeguard the health of the nation". As a result, the circulation of counterfeit drugs was reported to have been reduced by over 80% from what it was in 2001 [12], the amount of drugs unregistered by NAFDAC in circulation was reduced from 68% to 19%, and the production capacity of local pharmaceutical industries increased tremendously [13].

Prior to these reforms, the presence of counterfeit drugs had an obvious and detrimental impact on those who used them inadvertently. In 1990, 109 children died as a result of taking paracetamol syrup produced with toxic ethylene glycol instead of propylene glycol, a tragedy that occurred more than 50 years after its occurrence in the United States [14]. Despite NAFDAC's reported successes, examples of the spread and impact of counterfeit drugs in Nigeria still abound. In 2004, 3 Nigerian hospitals reported cases of adverse reactions from the use of contaminated infusions produced by 4 Nigerian companies [15]. It was confirmed that the infusions were heavily contaminated with microorganisms and 147 of the 149 brands of screened water for injection were found to be unsterile. Many of the other substandard drug products in Nigeria were/are present as a result of bad manufacturing practices and weak regulatory control [10,16].

This study assesses the level of transparency and potential vulnerability to corruption in four essential functions of Nigeria's pharmaceutical sector: registration, procurement, inspection, and distribution. **Registration **is the first of these decision points in any pharmaceutical system and is intended to ultimately guarantee a drug's efficacy in treating a specific disease and its safety profile. This process includes labeling, marketing, usage, warning and prescription requirements for the drug. As mentioned earlier, counterfeit drugs in Nigeria include those that were not registered with NAFDAC, those without active ingredients, expired drugs that were relabelled and resold, and drugs issued with incomplete manufacturer information [17]. Baseline studies conducted in 2001 showed that 68% of the drugs available in Nigeria were not registered with the Nigeria's National Agency for Food and Drug Administration and Control (NAFDAC) and later reports showed that as at 2004, counterfeit drugs still accounted for 40-50% of available drugs in Nigeria [18,19].

Procurement exists at the interface between the public system and drug suppliers. The purpose of procurement is to obtain the appropriate amount of drugs in the most cost-effective manner. Procurement involves managing inventory, aggregate purchasing, public bidding, analysis of offers, proper allocation of resources, payments, receipts of drugs purchased, and quality control checks [7].

Inspection of ports of entry and establishments such as local drug manufacturing sites is another crucial function of the Nigerian pharmaceutical sector. The majority of drugs in the country are imported from other countries in Asia such as India and China. Currently, there are 92 pharmaceutical companies producing only approximately 30% of Nigeria's internal drug supply [15]. Between 2001 and 2005, 30 Indian and Chinese pharmaceutical companies and 1 Pakistani company who were confirmed to be manufacturing counterfeit drugs were banned from exporting drugs to Nigeria [15]. Inspection of establishments occurs at the local and international level, where NAFDAC participates in ensuring Good Manufacturing Practices (GMP) of all establishments involved in the manufacture, sale, storage and distribution of drugs [20].

**Distribution **of drugs in the pharmaceutical sector involves allocating, transporting, and storing drugs appropriately at all times. Some medications have specific storage requirements such as refrigeration and secure facilities to minimize the risk of theft and other unethical practices. For this to occur, it is critical that information moves through each step of the system to control inventory and deliveries.

## Methods

The study was conducted from May to August 2007. The methodology is based on the WHO Assessment Tool of Medicines Regulatory Systems, 2007, which provided both qualitative and quantitative information on the level of transparency and susceptibility to corruption in each of the four functions of the public pharmaceutical sector: registration, procurement, inspection (of ports and establishments), and distribution. The assessment tool is currently being used in over 25 countries as part of the ongoing WHO Good Governance for Medicines Program.

Standardized questionnaires containing both open and closed-ended indicator questions adapted from the WHO assessment instrument [21,22] were used for each of the four functions. Fourteen semi-structured interviews were conducted in person by one of the study authors with key policy makers and stakeholders (furthermore referred to as *key informants*)representing both the public and private sector of the pharmaceutical system. Key informants included officials at NAFDAC and other government organizations, officials in non-government organizations, individuals who worked within the profession of pharmacy, and officers in judicial/law and order [see Additional File 1]. The key informants were selected purposefully according to their expertise and extent of involvement in each of the four functions, according to the relevance of their department to the area being researched, and based on their job description and institutional or corporate affiliation.

The questions for each function of the pharmaceutical system addressed key areas associated with a lack of transparency such as availability of information to the public, consistency in standard operating procedures, conflicts of interest, and accountability [see Additional File 2]. To minimize subjectivity, answers to closed-ended indicator questions were formulated to require a binary answer of yes or no. Responses were verified by hard data including written regulation and documented evidence of compliance with these regulations. A score of one (1) was given to "yes" responses that were validated by publicly available documentation. A score of zero (0) was given to "no" responses or "yes" responses without supporting documentation. A value of 1 represents lower vulnerability to corruption and a value of 0 represents higher vulnerability to corruption since the absence of a standardized process or documentation of such process creates inconsistencies in decision-making. For indicators with sub-questions, the total number of yes answers were tallied and divided by the total number of valid answers (an invalid answer is an answer of "don't know"). Table 1 illustrates this process.

**Table 1:** Example of tallying system for indicator questions with multiple sub-questions

Number of "yes" responses with evidence	6
Number of "no" responses or "yes" responses without evidence	2

Number of "don't know" responses	2

∴ Number of valid responses	8

**Scoring (total yes with evidence responses/total valid responses)**	**6/8 = 0.75**

Certain questions ask a respondent to what extent they agree to a statement. For such questions, the number of responses was tallied and the most frequent response was presented. The scale below was used to qualify the responses:

Strongly disagree

Disagree

Undecided

Agree

Strongly Agree

Not Applicable

Don't Know

Lastly, open ended questions were used for the purpose of obtaining additional information or perceptual information that could not be adequately collected via structured questions. The open ended questions also allowed key informants the opportunity to provide additional qualitative information that could help confirm findings from the closed ended questions or could also highlight areas that may not have been covered.

Upon completion of interviews and ratings of questions according to the criteria, the average rating was calculated for each function based on the WHO assessment instrument. The results were converted to a zero-to-ten (0.0 to 10.0) scale and were interpreted to represent the following incremental degrees of vulnerability to corruption [see Table 2].

**Table 2:** Numerical scale representing level of vulnerability to corruption

**0.0 - 2.0**	**2.1 - 4.0**	**4.1 - 6.0**	**6.1 - 8.0**	**8.1 - 10.0**
Extremely vulnerable	Very vulnerable	Moderately vulnerable	Marginally vulnerable	Minimally vulnerable

It is important to note that in pharmaceutical sector, or any other public system for that matter, identifying corruption is a not an easy task for a number of reasons. Firstly, individuals being interviewed may be reluctant to admit that corruption or any form of institutional weakness exists for fear of being directly implicated or for fear of reprimand. In addition, actual corruption and institutional inefficiency may be indistinguishable and often, inefficient systems can facilitate corruption or corruption can be hidden by inefficient systems [21]. Bearing this in mind, these questions and this scoring system are not intended to measure *actual *corruption. Rather, the point is to identify areas where potential *vulnerability *to corruption exists due to various deficiencies in governance and institutional weaknesses. Also noteworthy is the fact that the scores reflect the situation in the field at the time of the study. Since then, it is possible that steps may have been taken to address some of these deficiencies.

## Findings

The registration, procurement, inspection of ports, inspection of establishments, and distribution points of Nigeria's pharmaceutical system received scores of 5.8, 8.9, 6.4, 7.0, and 8.9 respectively, out of a maximum of 10 points. The overall scores for each function are summarized in Table 3. A more detailed summary is available in Table 4. According to the WHO assessment instrument used, these scores reflect a moderate vulnerability to corruption in the registration of drugs, marginal vulnerability to corruption in the inspection of ports, and establishments, and minimal vulnerability to corruption in the procurement and distribution of drugs. The overall rating was 7.4 out of 10, which indicates a marginal vulnerability to corruption in Nigeria's public pharmaceutical system. The overall rating was weakened by low scores in the area of drug registration and in the inspection of drugs at Nigeria's ports of entry.

**Table 3:** Summary of quantitative results measuring corruption in each section of Nigeria's pharmaceutical system

**Function**	**Score on a Scale of 1 to 10**	**Vulnerability to Corruption**
Registration	5.8	Moderate

Procurement	8.9	Minimal

Inspection of Ports	6.4	Marginal

Inspection of Establishments	7.0	Marginal

Distribution	8.9	Minimal

**OVERALL**	**7.4**	**Marginal**

**Table 4:** Detailed summary of results

**Area**	**Total Number of Indicator Questions**	**Score Obtained**	**Performance %**	**Score on a Scale of 10**
**Registration**	13	7.5	58%	**5.8**

**Procurement**	11	9.7	89%	**8.9**

**Inspection of Ports**	8	5.1	64%	**6.4**

**Inspection of Establishments**	8	5.5	70%	**7.0**

**Distribution**	13	11.6	89%	**8.9**

**TOTAL**	**53**	**39.4**	**74%**	**7.4**

## Discussion

### Registration

Drug registration is the responsibility of NAFDAC under the Registration and Regulatory Affairs Directorate [see Additional File 3 for an organogram of the subdivisions of NAFDAC]. Responsibilities of the directorate include the registration of drugs, foods and bottled water; auditing, monitoring and reporting on clinical trials; post-registration surveillance; and advertising control of regulated products [20]. Drug registration received the lowest score, an average rating of 5.8, indicating that this is the area most vulnerable to corruption in Nigeria's pharmaceutical sector.

#### Strengths

There is an up-to-date list of registered pharmaceutical products in the country which provides the minimum level of information about the products. Clearly documented procedures and standard forms exist and are publicly available for applications for drug registration. There is written documentation with well-defined standard operating procedures for assessors on how to process applications and there is a formal appeals process for applicants who have their applications rejected. A fixed committee composed of directors of various NAFDAC departments, directors within the Federal Ministry of Health, and officers of the Registration and Regulatory Affairs Directorate of NAFDAC assesses applications for drug registration.

#### Weaknesses

The rating given to drug registration was diminished by a combination of factors. There are no documented timeframes for processing applications as this has not yet been standardized. The only publically available timeline for registration processing is a 3 month timeframe indicated on the NAFDAC website [23].

There was no available documentation to support the selection and functioning of members of the committee that assesses applications. There was no documentation to support the requirement of any *specific *professional qualifications, technical skills, work experience, or research experience as criteria for membership of the committee. Documentation was lacking to describe the composition and terms of reference of the committee or the length of time that an individual could serve as a committee member. Also, guidelines for the committee's decision-making process as well as the decisions themselves were not required to be made publicly available. Written documentation that defines the committee's membership, composition, and terms of reference has been identified by the WHO's Good Governance for Medicines Programme as a tool to increase transparency and decrease vulnerability to corruption [1].

Guidelines to limit how and where registration officers meet with applicants were lacking and there were no written guidelines on conflict of interest. The only semblance of such guidelines was in the form of a declaration of assets which all civil servants complete upon commencement of employment with the government. This is vastly inadequate to address further conflicts of interests that may arise during the course of a registration officer's term. Screening of regulatory employees for conflicts of interest has been identified as a tool to lower the risk of corruption in the drug registration process and allow the regulatory authority to act impartially [2].

Bribery, offering of material gifts and favouritism occur commonly. Respondents reported that difficult living conditions in Nigeria sometimes lend officers to temptation. Indeed bribery is a common practice in Nigeria and has been identified as one of the leading causes of the rampancy of counterfeit medicines [22,9]. The process was so rampant that when Dr. Akunyili took over as Director General of NAFDAC, she fired a large number of NAFDAC officials [24]. However, each registration officer is not a sole decision maker therefore the potential for inducement may not affect decisions largely. Moreover, companies cannot readily alter lab results which are definitive criteria that determine approval for registration, limiting the extent of a company's influence on drug registration.

Respondents in community pharmacy cited the registration of patent and proprietary medicines dealers (non-pharmacists who are authorized to distribute specific over-the-counter medications outlined in the Essential Medicines List) as an avenue for infiltration of counterfeit drugs because many of these dealers operate in rural areas where regulation of their activities is difficult and many still sell medications that they are not permitted to sell [25].

Respondents also indicated that the registration process still does not address one of the root causes of counterfeiting in Nigeria: the fact that non-professionals (i.e. non-pharmacists) are still largely in control of retail pharmacy. It was reported that some pharmacies are owned by rich businesspersons who wish to cut corners and maximize profits by importing and selling substandard products. These individuals hire pharmacists as a front solely to obtain licensure to open and operate pharmacies. Using their wealth and influence, they often attempt to intimidate their way through normal regulatory procedures. At times, some of these syndicates are discovered and the individuals responsible are prosecuted, but the practice is apparently still prevalent [26].

### Procurement

Procurement of pharmaceuticals is under the mandate of Nigeria's Ministry of Health. The Ministry purchases a limited number of therapeutic classes of medications, namely anti-retrovirals, artemisin therapy for malaria, narcotics and controlled substances, vaccines, sulfadoxine, and anti-tuberculosis drugs. Procurement scored 8.9, indicating this area of the pharmaceutical sector has a low vulnerability to corruption. This is commendable particularly given the economic importance of this area and given that in many developing countries, drug procurement procedures are inefficient, non-transparent, and often corrupt [27]. This high score may be due to the fact that the Ministry procures a few therapeutic classes of drugs which do not account for a majority of total drug procurements.

#### Strengths

The process for procurement is competitive, documented, and well-defined (a concise manual on public procurement reform in Nigeria exists in print). Drugs are purchased using tenders (both international and local), except vaccines which are purchased through UNICEF or are donated. Objective methods are used to determine the quantity of pharmaceuticals needed using calculations that incorporate consumption data, population growth, projectional studies etc.

The criteria for membership and functions of the tender committee are clearly defined and are differentiated from that of the procurement office: the tender committee is made up of an Evaluation Committee and the Ministerial Tenders Board, which are responsible for evaluating supplier qualifications, inviting those who qualify to bid and determining which suppliers receive contracts. The procurement office, also known as the Budget Monitoring and Price Intelligence Unit (BMPIU) or the "Due Process" team, manages the tender process and finalizes the certificate of award and certificate of payment for the tender.

Procurement information is publicly available. Tenders are advertised in at least two national newspapers and in the Federal Tenders Journal. The bidding process and opening of applications for pre-qualification are also publicly accessible. Contracts are awarded to bidders who are pre-qualified and technically evaluated, but most importantly, who can execute the contract at the lowest cost. Some bidders, out of desperation, may quote prices unreasonably lower than fair market value. In such cases, contracts are awarded to bidders with the second lowest financial costs. There is a formal appeals process for applicants who have their bids rejected.

Management information systems to report problems in procurement exist in the form of written reports from the central medical stores where drugs are kept upon arrival. These include tracking records of the products ordered and delivered, records of quality assurance information, and evidence of communication between the procurement office and the central medical store when problems arise. A Monitoring and Evaluating Unit is tasked with reporting on the performance of suppliers, and works with the NAFDAC Inspectorate to ensure adequate packaging, labeling, potency, etc. of the products. Unsatisfactory performance has resulted in certain suppliers being blacklisted. Names of blacklisted companies are published routinely in public alert notices. Consignments are routinely inspected and samples analyzed upon inspection. The procurement process undergoes regular audits both internally (by the Internal Audit Unit of NAFDAC) and externally (by the Auditor General's office).

Bidders at times attempt to bribe procurement officers based on how they "sized them up". This type of activity was reported as being infrequent particularly because the procurement process is so open and because no single officer is a sole decision maker. In addition, more rigorous enforcement of regulations by NAFDAC has led to a higher calibre of bidding companies (prior to this, any local manufacturer had a shot at bidding regardless of the quality of their operations).

#### Weaknesses

As with drug registration, there is also a lack of written conflict of interest guidelines. This is concerning because the potential for corruption exists if members of the tender committee have vested interests in companies offering bids. Another weakness is a lack of evidence to suggest public availability of audit results. As discussed previously in registration, lacking conflict of interest guidelines and publicly available reports decreases transparency in the system and increases the vulnerability to corruption.

### Inspection of Ports

Nigerian ports of entry include airports, seaports and its national borders. The inspection of these ports offers unique operational challenges and multiple organizations undertake the activity. NAFDAC inspectors, present at all these ports, are assisted by the National Drug Law Enforcement Agency (NDLEA), the Nigerian Ports Authority (NPA), as well as other organizations. The decentralization of inspection, resulting in varying regulations and standards results in this area receiving a modest score of 6.4, the second lowest score of all the areas.

#### Strengths

Respondents indicated that the provision in the regulations for inspection at the ports is comprehensive, well-defined, and available to the companies involved in the importation of drugs. There are also well-defined written Standards of Practice (SOPs) for NAFDAC inspectors on how to conduct inspections. Guidelines for Good Manufacturing Practices (GMP) and Good Distribution Practices (GDP) are well-defined and are discussed in detail under Inspection of Establishments. The assistance from NDLEA and NPA inspectors at the ports further strengthens the ability of port authorities to discover irregularities. Collaborating with NAFDAC by providing both expertise and greater manpower also increases accountability for inspection findings.

#### Weaknesses

A principal weakness was the absence of written conflict of interest guidelines except for the incomprehensive "declaration of assets" form. Ports of entry are highly susceptible to the influx of counterfeit drugs and any laxity in vigilance of inspectors, which may be created by potential conflicts of interest, would seriously jeopardize progress in the battle against counterfeit pharmaceuticals.

Despite well-written regulations for port inspections, including well-defined SOP, respondents also highlight the prevalence of regulatory capture, the unethical practice where government officials who are supposed to act in the interest of the public are influenced by those that they are meant to be policing and engage in the very same unethical practices and behaviours that are supposedly being regulated [28]. The inconsistency in the written mechanisms or procedures to prevent regulatory capture between inspectors and suppliers thus leaves an open window for corrupt practices like the offering of bribes for inspectors to allow counterfeit products in.

According to respondents, anecdotes of bribery - the offer of material gifts, shares and investments in companies - are common at the ports. Companies have also been reported to become aggressive and threatening when deficiencies in their products are identified. Staffs at the ports are sometimes overwhelmed by the sheer volume of work and may inadvertently allow counterfeit drugs slip through, especially since there may be only one NAFDAC inspector at a time at a given port.

The NAFDAC claims that it cannot afford to have more than one inspector on the field, and there is heavy reliance on the presence of officials from other inspecting agencies to reduce strain. The propensity for regulatory capture is further exacerbated by the limited specificity in criteria needed for inspectors, including lack of specification of work experience or previous references in the field, an oversight which can result in employing inspectors who are inexperienced, less competent, and potentially more susceptible to inducement. The absence of any evidence of an internal review mechanism for the inspection procedure also causes concern, as it enables inefficiencies to be overlooked and allows counterfeit drugs to slip through the cracks.

### Inspection of Establishments

A country with high capital and sufficient infrastructure, Nigeria has a vibrant local drug manufacturing capacity. The role of NAFDAC in enforcing good manufacturing practices among local drug manufacturers is the inspection of establishments. Although improving under the current NAFDAC administration, inspection of establishment received a score of 7.0, indicating marginal vulnerability to corruption.

#### Strengths

Written guidelines that classify and define the Good Manufacturing Practices (GMPs) are available, outlining potential types of deficiencies and subsequent actions to be taken by the Inspectorate. A company may also seek an 'advisory inspection,' where a standard fee is paid to NAFDAC for advice from an inspector on GMP requirements and for recommendations of measures to improve GMP compliance at that site. To reinforce the emphasis on GMP, training programs for inspectors are routinely organized enabling them to travel overseas to countries where majority of manufacturing sites are compliant with GMP.

There are detailed SOPs and checklists for inspectors on how to conduct various kinds of inspections. Inspections are conducted in teams and are peer-reviewed under a well-structured chain of command. Inspectors (and sometimes re-inspections) are rotated on a scheduled system but inspectors remain within the same geographical area. The rationale for this is that inspectors can become more familiar with the idiosyncrasies of manufacturers in that geographical area, which increases their level of expertise. The process is subject to both an internal review and external audits. There is also a formal appeals process for companies who wish to contest the inspection findings however, claims must be supported by clear scientific evidence.

#### Weaknesses

Like the inspection of ports, the score for the inspection of establishments was hampered by the absence of written guidelines on conflict of interest. Respondents explained that conflicts of interest seldom truly affect the results of an inspection because inspections are conducted in teams of at least two individuals per site who undergo debriefing with a unit head and sign an inspection register after each inspection, thus no individual is the sole decision-maker. Written guidelines however, are necessary to ensure complete transparency as conflicts of interest can still exist in the presence of multiple checkpoints. Unless there are specific and comprehensive guidelines describing what constitutes conflict of interest and the process for controlling it, these conflicts will be addressed inconsistently or may even remain unaddressed, at the cost of transparency and accountability.

Inspectors at manufacturing sites are often presented with gifts from companies and there is an ill-defined demarcation between a "benign" offer of material goods and an attempt at inducement of an inspector by a company. Equally problematic is the fact that inspectors who visit manufacturing sites are transported there with vehicles from the company being inspected under what is referred to as a "partnership" with the companies. Not only is this a potential avenue for corruption, this poses a significant safety risk to the inspectors. Respondents have also indicated that it is not uncommon for inspectors to be impersonated. In addition, GMP guidelines are currently not publicly available but a committee is working on making them more accessible to companies being inspected.

The functions of the Inspectorate sometimes overlap with the Food and Drugs Department of the FMoH and the Pharmacists Council of Nigeria (PCN). The Inspectorate works with the Enforcement Directorate of NAFDAC to close down problematic sites including pharmacies. The PCN as the organization responsible for registering pharmacies used to have a major role in their inspection but NAFDAC has somewhat subsumed this responsibility and even has the right to shut down pharmacies. Respondents mentioned anecdotally that on some occasions, NAFDAC officers tend to be overzealous and have confiscated or destroyed legitimate drugs in addition to counterfeit ones. Some suggested that this was because not all officers are pharmacists and as such may not have detailed product knowledge.

### Distribution

Distribution of drugs received one of the highest scores with an average rating of 8.9 which indicates a low vulnerability to corruption. Distribution here refers to the movement of drugs procured by the government to the sites where they are needed (i.e. central medical stores, hospitals, etc.). The distribution of narcotics and psychotropic substances falls under the Narcotics and Controlled Substances Directorate of NAFDAC [20]. Retail distribution of drugs to pharmacies and private and proprietary medicines stores is not as well regulated.

#### Strengths

Inventory management models are adequate and are monitored regularly (usually weekly or at least monthly). Products are shelved according to therapeutic class and stock records are reconciled with physical counts weekly. Appropriate security management systems such as security personnel, restricted access to storage facilities, and monitored entry and exit of products are in place.

Audits are conducted yearly by the NAFDAC Internal Audit Unit, the Auditor General, and independent auditors. The system is monitored and evaluated on an ongoing basis by reviewing inventory records, return and disposal records etc. For vaccines, monitoring and evaluation is done quarterly by three parties: NAFDAC officials, UNICEF Vaccine Security Officers, and WHO Officers in each state. There is a fairly effective manual system to track the movement of drugs from warehouses to healthcare facilities, although the system may benefit from becoming computerized. Communication between distribution points is relatively effective, particularly given the popularity of GSM mobile telephones over the seldom functioning land lines in businesses, institutions, and government establishments.

#### Weaknesses

Security management systems could be strengthened by technology like alarm systems for security breaches and video cameras to monitor storage areas. Monitoring and evaluation are hindered by unexplained high staff turnover rates at municipal levels and the lack of adequate equipment for telecommunication in certain rural areas. Coding of government drugs could also be improved - aside from the NAFDAC registration number that all government-approved drugs are given, there is no special designation assigned to drugs purchased by the FMoH.

Rural areas pose a big challenge to drug distribution. Many lack electricity, well-established telecommunication networks, and certain technology like generators to provide backup power supply and WHO recommended fridges for the storage of thermolabile medications. As well, equipment is poorly maintained once supplied. Fuel scarcity also hinders the ability to transport drugs to more rural locations in a timely manner.

The retail distribution of drugs has been described as chaotic [29] and is considered virtually unregulated. Pharmacies and private and proprietary medicines stores are able to procure drugs in bulk from local drug manufacturers [30]. In addition, prescription and non-prescription drugs are sold on the open market [31]. This creates an entry point for counterfeit medicines into the pharmaceutical market.

## Recommendations

Optimism about the reform process led by NAFDAC is clear amongst the respondents. While a general sense of progress was expressed by most, it took very little incitement to receive a multitude of recommendations for improvement among all four facets of the Nigerian pharmaceutical system. The recommendations are summarized in Table 5 and are categorized according to their importance and feasibility. The higher priority recommendations are discussed below.

**Table 5:** Prioritization of Recommendations*

	**High Feasibility**	**Low Immediate Feasibility**
**High Priority**	• Establish clear and well documented conflict of interest (COI) guidelines • Define penalties for infringement of COI guidelines and enforce them consistently • Make internal and external audits of drug regulatory agencies publicly available • Monitor of patent and proprietary medicines dealers more closely • Make terms of reference and criteria for membership of the drug registration committee publicly available	• Re-allocation of financial resources to provide more up-to-date technology • Increase security (preferably by electronic alarm systems, video cameras etc) at drug storage facilities and warehouses • Implementing incentives to favour high-quality locally manufactured drug products

**Low Priority**	• Regular rotation of inspectors to different geographical locations • Decentralize NAFDAC inspectorate offices within states and increase the number of local government offices	• Potential eradication of the sale of drugs by non-pharmacists • Improve the division of responsibility for drugs among the various departments of the Federal Ministry of Health

*The classification of the feasibility was determined based on estimates of financial or human resource constraints and availability of infrastructure necessary to implement these recommendations.

The most pervasive institutional weakness identified from this study was the lack of explicitly documented conflict of interest guidelines for any of the four functions of the pharmaceutical sector. Many other weaknesses stemmed from this deficiency. Thus, the primary recommendation would be to establish clear and well-documented conflict of interest (COI) guidelines for personnel of NAFDAC, the National Drug Law Enforcement Agency (NDLEA), and other public officials in the pharmaceutical sector. When conflict of interest guidelines are present and consistently administered, the promulgation of corrupt practices may be limited. Nigeria can model its COI guidelines after those of public drug systems of other countries, for example, the conflict of interest guidelines for the Common Drug Review by the Canadian Agency for Drugs and Technologies in Health [32]. In addition to developing COI guidelines, the Nigerian government also needs to ensure consistent application of the guidelines, which may involve the establishment of penalties for breaching COI principles. This would be a relatively low cost initiative to implement and one that has potential to result in some significant improvements in the system.

The second most important recommendation is for better allocation of financial and other resources by NAFDAC and investing in up-to-date technology to assist officers in their work. Purchasing vehicles for inspectors of would halt the practice of having manufacturing companies transport inspectors to and fro manufacturing sites. This would not only reduce the safety risk to the inspectors, it would also limit the potential for enticement and regulatory capture. Purchasing video cameras and electronic alarm systems for drug storage sites prior to distribution would greatly enhance security and accountability for procured products. However, it is acknowledged that the greater problem of the nation's erratic power supply may impede the immediate implementation of an electronic security system. Acquiring camcorders and other battery-operated devices to aid remote inspections and on-the-spot testing of drugs would increase the accuracy of the inspection process. Purchasing mobile GSM phones for officers in the field or on the road would greatly improve communication between departments. More modern laboratory equipment would also enable better detection of counterfeit drugs. Identifying the most worthwhile ventures to allocate investments into can be determined by regular diagnostics of the pharmaceutical system.

A third recommendation is to place greater emphasis on human resources. This involves hiring qualified personnel and re-training current staff. Short staffing was a particular problem in drug inspection at the ports and this is problematic given that the ports are a porous entry point for counterfeit drugs and with fewer staff, more counterfeit products are likely to be missed. In addition, an enhanced salary and benefit package for current NAFDAC officers would help shield them from tempting offers made by desperate companies, however, this would be contingent on an increase in budgetary allocation to the NAFDAC from the Federal Government.

Fourthly, the local pharmaceutical industry may benefit from incentives which favour registration of high-quality locally manufactured drug products over foreign imports. One of NAFDAC's mandates is to "strengthen confidence in made-in-Nigeria products" so processes that favour local drug manufacturers in the long term could result in a more responsive and accountable industry which ensures higher quality of locally-made products and would also stimulate the nation's economy. The establishment of such initiatives would require initial investments into Nigeria's manufacturing facilities so that they are in line with WHO Good Manufacturing Practices as a starting point.

To further restore confidence in the Nigerian drug industry, closer monitoring of patent and proprietary medicines dealers is recommended to ensure that they do not distribute drugs they are not authorized to. This could be in the form of both routinely scheduled and impromptu inspections of their facilities. As well, the suppliers of their products should be verified and more closely monitored by inspectors to ensure that they are legitimate, compliant with NAFDAC regulations, and that their products are quality controlled. Some respondents went as far as calling for the abolishment of sale of drugs by non-pharmacists altogether. The immediate feasibility of the latter is questionable, however, given that many rural areas in Nigeria do not have pharmacists or even any formally-trained health care professionals.

A sixth recommendation is for public availability of internal/external audit results of drug regulatory agencies. Apprehension about public scrutiny may provide an incentive for all agencies to ensure that their processes are well-documented and consistent, and that they are fully compliant with the standard operating procedures set out for them. This measure would increase transparency as well. Auditors may benefit from occasional collaborations with foreign partners such as the WHO, the United States Food and Drug Administration, and Health Canada, among others, thus increasing the validity, reliability, and thoroughness of audits and providing a means for external monitoring and evaluation of the audit process. In accordance with the theme of public accountability, the area of drug registration suffered from a lack of public availability of documentation describing the terms of reference or criteria for member selection of the committee that decided which drugs would be approved for registration. Being able to assess these documents publicly would also improve transparency and with increased transparency, there is a lower potential for inefficiency and/or corruption [21].

To reinforce transparency and accountability of the inspection process, it is also recommended to regularly rotate inspectors to different sites. An explanation given for not rotating inspectors was that greater lengths of time spent at a particular port increased the expertise of the inspectors there. Furthermore, respondents demanded that some inspection units should be decentralized into the various local government areas as opposed to remaining primarily in state capitals. This places inspectorate offices much closer to their jurisdictions and this will quicken response times as well as reduce inefficiencies created by gaps in communication between local, state, and federal bureaucracies. The establishment of inspectorate offices in local government areas is in fact part of a 10 year rolling plan of NAFDAC.

### Study Limitations and Recommendations for Further Research

Acquiring empirical evidence is difficult when researching corruption particularly because of reluctance to disclose information that may portray a particular government or organization as inefficient. This methodology, which has been field-tested and revised by the WHO, attempts to quantify information that is usually very difficult to quantify, and while it does not directly measure corruption itself, it serves the more useful purpose of identifying areas where key informants perceive that there are vulnerabilities to corruption in a consistent and systematic manner. The methodology is being continuously refined to ensure it depicts the situation more accurately in countries under review. There were, however, certain limitations to our study.

Firstly, obtaining appointments with key informants was difficult and certain stakeholders were reluctant to be interviewed, which is expected given the nature of the issue being studied. The study was undertaken during federal elections and a change of government which made it even more difficult to recruit informants or secure appointments as many public organizations also underwent personnel changes, staff went on holidays, and there were scheduling conflicts.

Other events such as a fuel crisis and a national labour union strike stalled progress and increased the time constraint during the period of data collection.

Secondly, having more interviewers in the field would have quickened the information gathering process. However this was not a significant problem as the interviewer is a Nigerian national and was familiar enough with the environment to navigate it well.

Thirdly, the methodology does not fully capture the inefficiencies *outside *of government which perpetuate the misuse of drugs such as the impersonation of NADFAC officers, counterfeiting of NAFDAC forms, the sale of drugs by unauthorized dealers, dispensing of prescription drugs like antibiotics *without *a prescription, and the control of pharmacies by non-pharmacists. Nigeria would benefit from further research to ascertain the magnitude of these challenges and means to address them. This methodology was largely a tool to gauge the perceptions of key policy makers and stakeholders who have a vested interest in the state of the pharmaceutical system. Perception and reality are not necessarily commensurate. There is still value in this sort of study as an effort to catalyze an investigation into the problem. In examining what the current perceptions are, the door is opened for further research into some of the discrepancies between these perceptions and the reality of the situation.

Fourthly, given the sample size, there is the potential for bias and error, as with any qualitative study. However, field experience using this methodology has shown that ten to fifteen interviews for each function of the pharmaceutical system are optimal although there may be some variance depending on the situation and area of the pharmaceutical sector being studied [21]. The primary goal is to interview enough people until saturation of themes is reached i.e. there is a recurrence/repetition of concepts and little or no new information is gleaned. The potential for error was also reduced by only accepting the quantitative responses as valid if there was hard evidence or documentation to verify the claims. For example, one of the questions under drug registration asked if there was a formal appeals system for those whose applications for registration were rejected. If the response was "yes", a score would only be given if the investigator provided evidence that such an appeals process existed i.e. by showing the application form itself, by documenting meeting minutes of the review process etc.

Lastly, given that Nigeria has made a number of advances in drug regulation over the last six years, it may have been beneficial to do a "before/after" study i.e. to conduct such the same kind of study when NAFDAC began its reforms in 2001 and then conduct a subsequent assessment. This may have provided beneficial information on which areas showed the greatest improvement and which areas still lag behind. Such comparative information can still be gathered by repeating this study in a few years time.

## Conclusion

Global attention to the battle against counterfeit drugs is increasing. The WHO fact sheet on counterfeit medicines refers to them as "an enormous public health challenge" and reports that drug counterfeiting is the greatest in regions with legal oversights and weaknesses in regulatory control of pharmaceuticals [33]. The fact sheet predicts a greater than 90% increase in counterfeit drug sales by the year 2010.

Despite this global trend, Nigeria stands out as a country that has acknowledged its problem and made some degree of progress in dealing with counterfeit drug production and trafficking. Much this success can be attributed to stronger leadership and political will within NAFDAC and greater public awareness of the problem. However, there is still much work to be done. Greater institutional robustness and appropriate checks and balances are necessary to ensure a sounder, more effective, and more transparent pharmaceutical system in Nigeria. These study findings and recommendations will hopefully enable Nigeria evaluate the operating structure of their pharmaceutical sector, capitalize on its strengths, and institute necessary reforms to address its deficiencies.

## Abbreviations

COI: Conflict of Interest; FMoH: Federal Ministry of Health; GMPs: Good Manufacturing Practices; NAFDAC: National Agency for Food and Drug Administration and Control; NDLEA: National Drug Law Enforcement Agency; NPA: Nigerian Ports Authority; PCN: Pharmacists Council of Nigeria; SOP: Standard Operating Procedure.

## Competing interests

The authors declare that they have no competing interests. Jillian Clare Kohler has worked as a consultant for the World Health Organization on good governance issues.

## Authors' contributions

HAG conceived of the study, participated in its design, carried out the field interviews with key stakeholders, drafted the manuscript, generated and refined the study analysis and outlined the conclusions. JCK coordinated the study, narrowed the initial study focus, participated in the design of the study, developed the methodology directed the research project, vetted the manuscript, refined the analysis/discussion of results and solidified the conclusions. AMH drafted and reviewed the manuscript, contributed to the final analysis and fine-tuned the study conclusions. All authors read and approved the final manuscript.

## Supplementary Material

Additional file 1 **List of key informants.** Click here for file

Additional file 2 **Questionnaire.** Click here for file

Additional file 3 **Organogram of the subdivisions of NAFDAC.** Click here for file
