# Supplementary material for: Transparency in Nigeria's public pharmaceutical sector: perceptions from policy makers
Source: Global Health. 2009 Oct 29;5:14. doi: 10.1186/1744-8603-5-14 (PMC2775729; doi:10.1186/1744-8603-5-14)
Supplement: Additional file 1 — List of key informants. [file 1744-8603-5-14-S1.doc]

# Transparency in Nigeria’s Public Pharmaceutical Sector: Perceptions from Policy Makers

*Habibat A Garuba, Jillian C Kohler, Anna M Huisman*

### Additional File 1

### Title: List of Key Informants (Interview Participants)

**Description:** A list of the interviewed subjects and their position within the organizations that they represent.

1. Director of NAFDAC Administration & Finance Directorate
2. Assistant Regulatory Officer in the Pharmacovigilance/Food and Drug Information Unit
3. Assistant Chief Regulatory Officer in the Establishment Inspection Directorate (NAFDAC)
4. Deputy Director in the Ports Inspection Directorate (NAFDAC)
5. Chief Regulatory Officer; Drug Regulation and Registration (NAFDAC)
6. Chief Regulatory Officer in the Narcotics and Controlled Substances Directorate
7. Director of Vaccine Security and Logistics (National Primary Health Care Development Agency (NPHCDA – Department of Immunization)
8. Director of Finance & Administration (Axios Foundation/GhAIN)
9. Executive Director of Diamond Remedies (Pharmaceutical Company)
10. Deputy Director of Drugs and Vaccine Development – Federal Ministry of Health, Dept of Food and Drugs
11. Principal Health Planning Officer – Federal Ministry of Health, Dept of Health Planning and Research
12. H.O.D. Pharmacy, State House Medical Centre
13. Clinical Pharmacist (PharmD), State House Medical Centre
14. H.O.D. Pharmacy, National Hospital
15. Community Pharmacists – Skylark Pharmacy
16. Community Pharmacists - Therapeutics Pharmacy
