# Supplementary material for: Transparency in Nigeria's public pharmaceutical sector: perceptions from policy makers
Source: Global Health. 2009 Oct 29;5:14. doi: 10.1186/1744-8603-5-14 (PMC2775729; doi:10.1186/1744-8603-5-14)
Supplement: Additional file 2 — Questionnaire. [file 1744-8603-5-14-S2.docx]

# Transparency in Nigeria’s Public Pharmaceutical Sector: Perceptions from Policy Makers

*Habibat A Garuba, Jillian C Kohler, Anna M Huisman*

**Additional File 2**

**Title:** Questionnaires and scores

**Description:** The data provided represent the questionnaires administered to the key informants for each of the following pharmaceutical functions:

1. Drug Registration
2. Procurement
3. Inspection of Ports
4. Inspection of Establishments
5. Drug Distribution

**I. DRUG REGISTRATION**

| ***Question*** | | ***Criteria*** | |
| --- | --- | --- | --- |
| 1 | *Is there an up to date list of all registered pharmaceutical products available in the country?* | Yes/No | 1 |
|  |  | Percentage Correct | 100% |
|  |  | Score | 1 |
|  | | | |
| 2 | *If such a list exists, does it provide a minimum level of information?* | Product description (name: brand & INN, dosage form, strength, primary packaging, any identifying mark  Name of manufacturer  Country of manufacture  Site of manufacture  Date of registration  Validity of registration  Conditions for registration  Prescription only or OTC | 1  1  1  1  1  1  1  1 |
|  |  | Percentage Correct | 100% |
|  |  | Score | 1 |
|  | | | |
| 3 | *Are there written procedures for applicants on how to submit an application for registration of medicinal products?* | Written procedures  Publicly accessible  Describe the process to follow in submitting an application  Mention timeframe for processing  Mention fees  Mention data to be submitted  Mention criteria for registration | 1  1  1  0  1  1  1 |
|  |  | Percentage Correct | 86% |
|  |  | Score | 0.86 |
|  | | | |
| 4 | *Are there written procedures for assessors on how to assess applications submitted for registration of medicinal products?* | Written procedures  Publicly accessible  Describe the process to follow in assessing submissions  Mention timeframe for processing  Specify issues to be considered in assessing submissions  Provide guidance on report writing | 1  0  1  0  1  1 |
|  |  | Percentage Correct | 67% |
|  |  | Score | 0.67 |
|  | | | |
| 5 | *Is there a standard application form publicly available for submission of application for registration of medicinal products?* | Publicly accessible  Readily available at government office  Requires description of the product: name of product (brand name & INN), composition per unit dose  Brief summary of method of manufacture  Specification of pharmaceutical ingredients and excipients  Summary Product Characteristics (SPC): Pharmacological action, therapeutic classification, indications, contraindications, etc.  Packaging material and inserts  Labelling | 1  1  1  1  1  1  1  1 |
|  |  | Percentage Correct | 100% |
|  |  | Score | 1 |
|  | | | |
| 6 | *Are there written guidelines setting limits on how and where medicines registration officers meet with applicants?* | Yes/No | 0 |
|  |  | Percentage Correct | 0% |
|  |  | Score | 0 |
|  | | | |
| 7 | *Is there a functioning formal committee responsible for assessing applications for registration of pharmaceutical products?* | Yes/No | 1 |
|  |  | Percentage Correct | 100% |
|  |  | Score | 1 |
|  | | | |
| 8 | *Are there clear written criteria for selecting the members of the committee?* | Written criteria  Criteria publicly available  Specify professional qualification required  Specify the technical skills and work experience related to the area  Require declaration of conflict of interest (e.g. investment in pharmaceutical business)  Require research experience in the area of expertise  Specify organization to be considered when selecting members  Give a timeframe to serve as a committee member | 1  1  0  0  0  0  0  0 |
|  |  | Percentage Correct | 25% |
|  |  | Score | 0.25 |
|  | | | |
| 9 | *Is there a written document that describes the composition and terms of reference of the committee?* | Up-to-date document  Publicly accessible  Includes names of the members  Includes duties, responsibilities and obligations of the members  Includes the accountability of the members  Includes quorum requirement  Includes membership terms/rotation requirements  Includes the financial benefits of the members, if any | 0  0  0  0  0  0  0  0 |
|  |  | Percentage Correct | 0% |
|  |  | Score | 0 |
|  | | | |
| 10 | *Are there written guidelines on conflict of interest (COI) with regard to registration activities?* | Guidelines on COI exist in writing  Form for declaration of COI for members of registration committee exists  Include rules on the acceptance of gifts  Include rules on reporting conflict of interest  Include a mechanism protecting informers of COI  Include actions to be taken in case of failure to comply with policy  Evidence of enforcement of these regulations | 0  0  0  0  0  0  0  0 |
|  |  | Percentage Correct | 0% |
|  |  | Score | 0 |
|  | | | |
| 11 | *To what extent do you agree with the following statement: “The members of the registration committee are systematically and objectively selected based on the written criteria in force in your country?”* | Strongly disagree |  |
|  | | | |
| 12 | *Are there clear and comprehensive guidelines for the committee’s decision making process?* | Describe clearly the mandate of the committee  Available publicly and in writing  Describe the number of meetings it should convene  Describe procedures for decision-making  Include clear time limits for decision-making process for the committee  Describe the reporting structure  Decisions of meetings need to be publicly available | 1  0  1  1  1  1  0 |
|  |  | Percentage Correct | 71% |
|  |  | Score | 0.71 |
|  | | | |
| 13 | *Is there a formal appeals system for applicants who have drug applications rejected?* | Yes/No | 1 |
|  |  | Percentage Correct | 100% |
|  |  | Score | 1 |
|  | | | |
| 14 | *To what extent do you agree with the following statement: “Gifts and other benefits given to the officials in charge of medicines registration have no influence at all on the final decisions?* | Strongly agree |  |
|  | | | |

**II. PROCUREMENT OF DRUGS**

| ***Question*** | | ***Criteria*** | |
| --- | --- | --- | --- |
| 1 | *Does the government use transparent and explicit procedures for procurement of pharmaceutical products?* | Written procedures publicly available  Describe the internal process to be followed by staff on how to process the bids  Require the use of generic names  Require procurement to be based on the national essential medicines list  Require advertisement of tenders  Require that contract specifications be publicly available  Require that criteria for adjudication of tender be included as part of the tender package  Require that contract awards be made by the tender committee  Require that information on tender process and results are made public (to the extent permitted by law) | 1  1  1  1  1  0  1  1  1 |
|  |  | Percentage correct | 89% |
|  |  | Score | 0.88 |
|  | | | |
| 2 | *Is there written guidance for procurement office staff on the type of procurement method to be used for different types of products?* | Yes/No | 1 |
|  |  | Percentage correct | 100% |
|  |  | Score | 1 |
|  | | | |
| 3 | *Is procurement done with an objective quantification method to determine the quantity of pharmaceuticals to be purchased?* | Yes/No | 1 |
|  |  | Percentage correct | 100% |
|  |  | Score | 1 |
|  | | | |
| 4 | *Is there a formal appeals process for applicants who have their bids rejected* | Yes/No | 1 |
|  |  | Percentage correct | 100% |
|  |  | Score | 1 |
|  | | | |
| 5 | *Is there a tender committee (TC)? If so, are the key functions of the procurement office and those of the tender committee clearly separated?* | There is a TC formally established  TC responsible for suppliers’ selection for restricted tenders  TC responsible for contract decisions | 1  1  1 |
|  |  | Percentage correct | 100% |
|  |  | Score | 1 |
|  | | | |
| 6 | *Are there specific criteria for tender committee membership?* | Criteria publicly available  Criteria clearly written  Require professionals with specific functions or skills  Require representation from senior government officials  Require representation from client facilities  Require that membership changes periodically  Require that members declare COI | 1  1  1  1  1  1  0 |
|  |  | Percentage correct | 100% |
|  |  | Score | 1 |
|  | | | |
| 7 | *Are there written guidelines on conflict of interest (COI) with regard to the procurement process* | Guidelines on COI exist in writing  Form for declaration of COI for members of tender committee exists  Includes rules on the acceptance of gifts  Includes rules on reporting conflict of interest  Includes a mechanism protecting informers of COI  Include actions to be taken in a case of failure to comply with policy  Evidence of enforcement of these regulations | 0  0  0  0  0  0  0 |
|  |  | Percentage Correct | 0% |
|  |  | Score | 0 |
|  | | | |
| 8 | *To what extent do you agree with the following statement: “the members of the tender committee are systematically selected based on specific criteria (see question 6)?* | Agree |  |
|  | | | |
| 9 | *Is there a computerized management information system used to report product problems in procurement?* | Management information system exists  Includes product records  Monitors suppliers performance  Monitors facilities (clients) performance  Records quality assurance information  Tracks status for each order  Tracks quantities purchased compared with estimates | 1  1  1  1  1  1  1 |
|  |  | Percentage Correct | 100% |
|  |  | Score | 1 |
|  | | | |
| 10 | *Are there standard operating procedures (SOPs) for routine inspection of consignments* | Each shipment physically checked  Samples taken and sent to quality control labs randomly for all consignments  Samples taken and sent to quality control labs systematically for new suppliers  Inspections reported in documents and archived in the procurement office | 1  1  1  1 |
|  |  | Percentage Correct | 100% |
|  |  | Score | 1 |
|  | | | |
| 11 | *Is there an efficient post-tender system in place to monitor and report on suppliers’ performance to the tender committee?* | Supplier’s performance monitored at least annually  Monitoring system tracks suppliers lead-time  Monitoring system tracks the shelf-life  Monitoring system tracks the packaging of products  Procurement agency has a list of previous suppliers  Suppliers with poor performance are identified as blacklisted | 1  1  1  1  1  1 |
|  |  | Percentage Correct | 100% |
|  |  | Score | 1 |
|  | | | |
| 12 | *Does the procurement office undergo regular audits?* | Audit is compulsory by law  Done on an annual basis  Results publicly available  Audit conducted by an independent unit (internal or external)  Reports operating costs of procurement office  Reports pharmaceutical products tendered  Reports quantities of the products  Reports the beneficiaries | 1  1  0  1  1  1  1  1 |
|  |  | Percentage Correct | 88% |
|  |  | Score | 0.88 |
|  | | | |
| 13 | *To what extent do you agree with the following statement: “The procurement system in your country is operating in a totally transparent manner”?* | Agree |  |
|  | | | |

**III. INSPECTION OF PORTS**

| ***Question*** | | ***Criteria*** | |
| --- | --- | --- | --- |
| 1 | *Is there a provision in the medicines legislation/regulation covering inspection of medicines and manufacturers and distributors?* | Yes/No | 1 |
|  |  | Percentage Correct | 100% |
|  |  | Score | 1 |
|  | | | |
| 2 | *Is the provision comprehensive enough?* | Provides power to inspectors to enter at any reasonable time any place where medicinal products are product, packaged, stored, distributed, or tested.  Defines the inspectors duties and responsibilities  Provides special identification document to the inspectors  Provision is available to companies being inspected | 1  1  1  1 |
|  |  | Percentage Correct | 100% |
|  |  | Score | 1 |
|  | | | |
| 3 | *Are there written guidelines on classification of Good Manufacturing Practices (GMP) or Good Distribution Practices (GDP) non-compliance that describe the types of deficiencies and the corresponding measures to be taken by the Medicine Regulatory Authority?* | Guidelines available in writing  Guidelines provide classification of GMP/GDP  Define corresponding measures to be taken in case of non-compliance  Guidelines easily accessible to all stakeholders  Provide appeals mechanism for companies  Appeals system independent of the body making the original decision | 1  1  1  1  1  0 |
|  |  | Percentage Correct | 83% |
|  |  | Score | 0.83 |
|  | | | |
| 4 | *Are there written procedures/mechanisms to prevent regulatory capture b/w inspectors and the manufacturers or distributors that he/she inspects?* | Procedures available in writing  Require rotation of inspectors based on scheduling system  Require inspectors to visit sites in teams with a team leader  Require inspectors to inspect under the observation of another inspector who will report on what he/she has observed (peer review)  Rotation mechanism requires inspectors from one geographical area to inspect ports in other areas  Require external audit of the inspections (from another country) | 1  0  1  1  1  0 |
|  |  | Percentage Correct | 67% |
|  |  | Score | 0.67 |
|  | | | |
| 5 | *Are there written guidelines on conflict of interest (COI) with regard to inspection activities?* | Guidelines on COI exist in writing  Form for declaration of COI for inspectors exists  Include rules on acceptance of gifts  Include rules on reporting COI  Include mechanism protecting informers of COI  Include actions to be taken in case of failure to comply with the policy  Evidence of enforcement of these regulations | 0  1  0  0  0  0  0  0 |
|  |  | Percentage Correct | 14% |
|  |  | Score | 0.14 |
|  | | | |
| 6 | *Are inspection findings and conclusions subject to an internal review?* | Yes/No | 0 |
|  |  | Percentage Correct | 0% |
|  |  | Score | 0 |
|  | | | |
| 7 | *Are there written standard operating procedures (SOPs) for inspectors on how to conduct inspections?* | An inspection checklist/aide-memoire  Procedures detailing requirements for pre-inspection  Procedures detailing requirements for post-inspection activities  Scheduling system identifying companies due for inspections within a set time-frame  Format and content of inspection reports | 1  1  1  1  1 |
|  |  | Percentage Correct | 100% |
|  |  | Score | 1 |
|  | | | |
| 8 | *Are there written criteria for the selection and recruitment of inspectors? If yes, do they include the following:* | Available in writing and publicly  Qualification required (pharmacist, chemist, etc)  Minimum years of work experience in the area  Recommendation from (past workplace, association) | 1  1  0  0 |
|  |  | Percentage Correct | 50% |
|  |  | Score | 0.5 |
|  | | | |

**IV. INSPECTION OF ESTABLISHMENTS**

| ***Question*** | | ***Criteria*** | |
| --- | --- | --- | --- |
| 1 | *Is there a provision in the medicines legislation/regulation covering inspection of medicines and manufacturers and distributors?* | Yes/No | 1 |
|  |  | Percentage Correct | 100% |
|  |  | Score | 1 |
|  | | | |
| 2 | *Is the provision comprehensive enough?* | Provides power to inspectors to enter at any reasonable time any place where medicinal products are product, packaged, stored, distributed, or tested.  Defines the inspectors duties and responsibilities  Provides special identification document to the inspectors  Provision is available to companies being inspected | 1  1  1  0 |
|  |  | Percentage Correct | 100% |
|  |  | Score |  |
|  | | | |
| 3 | *Are there written guidelines on classification of Good Manufacturing Practices (GMP) or Good Distribution Practices (GDP) non-compliance that describe the types of deficiencies and the corresponding measures to be taken by the Medicine Regulatory Authority?* | Guidelines available in writing  Guidelines provide classification of GMP/GDP  Define corresponding measures to be taken in case of non-compliance  Guidelines easily accessible to all stakeholders  Provide appeals mechanism for companies  Appeals system independent of the body making the original decision | 1  1  1  0  1  0 |
|  |  | Percentage Correct | 67% |
|  |  | Score | 0.67 |
|  | | | |
| 4 | *Are there written procedures/mechanisms to prevent regulatory capture b/w inspectors and the manufacturers or distributors that he/she inspects?* | Procedures available in writing  Require rotation of inspectors based on scheduling system  Require inspectors to visit sites in teams with a team leader  Require inspectors to inspect under the observation of another inspector who will report on what he/she has observed (peer review)  Rotation mechanism requires inspectors from one geographical area to inspect companies in other areas  Require external audit of the inspections (from another country) | 1  1  1  1  0  0 |
|  |  | Percentage Correct | 67% |
|  |  | Score | 0.67 |
|  | | | |
| 5 | *Are there written guidelines on conflict of interest (COI) with regard to inspection activities?* | Guidelines on COI exist in writing  Form for declaration of COI for inspectors exists  Include rules on acceptance of gifts  Include rules on reporting COI  Include mechanism protecting informers of COI  Include actions to be taken in case of failure to comply with the policy  Evidence of enforcement of these regulations | 0  D.K.  0  0  0  D.K  0 |
|  |  | Percentage Correct | 0% |
|  |  | Score | 0 |
|  | | | |
| 6 | *Are inspection findings and conclusions subject to an internal review?* | Yes/No | 1 |
|  |  | Percentage Correct | 100% |
|  |  | Score | 1 |
|  | | | |
| 7 | *Are there written standard operating procedures (SOPs) for inspectors on how to conduct inspections?* | An inspection checklist/aide-memoire  Procedures detailing requirements for pre-inspection  Procedures detailing requirements for post-inspection activities  Scheduling system identifying companies due for inspections within a set time-frame  Format and content of inspection reports | 1  1  1  1  1 |
|  |  | Percentage Correct | 100% |
|  |  | Score | 1 |
|  | | | |
| 8 | *Are there written criteria for the selection and recruitment of inspectors? If yes, do they include the following:* | Available in writing and publicly  Qualification required (pharmacist, chemist, etc)  Minimum years of work experience in the area  Recommendation from (past workplace, association) | 1  1  0  0 |
|  |  | Percentage Correct | 100% |
|  |  | Score | 1 |
|  | | | |

**V. DISTRIBUTION CRITERIA**

| ***Question*** | | ***Criteria*** | |
| --- | --- | --- | --- |
| 1 | *Is there an inventory management model that is used?* | Addresses: amount of safety stock  Reorder frequency  Reorder quantity  Includes: limited access to stores  Store locks  Record keeping and stock level monitoring | 1  1  1  1  1  1 |
|  |  | Percentage correct | 100% |
|  |  | Score | 1 |
|  | | | |
| 2 | *Are inventory levels monitored on at least a monthly basis?* | Monitored on at least a monthly basis  Written or computerized records of inventory levels | 1  1 |
|  |  | Percentage correct | 100% |
|  |  | Score | 1 |
|  | | | |
| 3 | *Are stock records reconciled with the physical counts on a regular basis?* | Reconciled on a regular basis | 1 |
|  |  | Percentage correct | 100% |
|  |  | Score | 1 |
|  | | | |
| 4 | *Is there systematic and orderly shelving of drug products in warehouses?* | Drugs are stocked by therapeutic category or alphabetically | 1 |
|  |  | Percentage correct | 100% |
|  |  | Score | 1 |
|  | | | |
| 5 | *Is there a security management system in place to oversee storage and distribution* | Monitoring of entry and exist to warehouses  Limited access  Alarm system for security breaches  Security cameras  Security force or responsible individuals | 1  1  0  0  1 |
|  |  | Percentage correct | 60% |
|  |  | Score | 0.6 |
|  | | | |
| 6 | *Are there independent audits of warehouses by national inspectors or outside contractors?* | Clear and comprehensive  Performed by independent persons  Regular audits | 1  1  1 |
|  |  | Percentage correct | 100% |
|  |  | Score | 1 |
|  | | | |
| 7 | *Are sanctions imposed on individuals for theft or corrupt practices?* | Evidence of sanction enforcement | 0 |
|  |  | Percentage Correct | 0% |
|  |  | Score | 0 |
|  | | | |
| 8 | *Has any public official been dismissed in the past two years for unethical behaviour? (i.e. theft; corrupt practices)* | Evidence of sanctions imposed for corrupt practices | 1 |
|  |  | Percentage Correct | 100% |
|  |  | Score | 1 |
|  | | | |
| 9 | *Is there an information system (computerized or manual) to track the movement of pharmaceuticals from a warehouse to a health facility* | Type of drugs left at warehouse  Amount of drugs left at warehouse  Intended recipient of drugs  Time and date of drug arrival | 1  1  1  1 |
|  |  | Percentage Correct | 100% |
|  |  | Score | 1 |
|  | | | |
| 10 | *Is there coding used to identify government drugs?* | Clear and effective coding system | 0 |
|  |  | Percentage Correct | 100% |
|  |  | Score | 1 |
|  | | | |
| 11 | *Is there a formal process to verify that the drugs received by the provider match those shipped from the warehouse or the supplier?* | Receipts are checked against the packing slip | 1 |
|  |  | Percentage Correct | 100% |
|  |  | Score | 1 |
|  | | | |
| 12 | *Does a program exist for monitoring and evaluating the performance of the drug distribution system?* | Performance is evaluated on a regular basis  Evaluated by an independent authority  Identified weaknesses are addressed | 1  1  1 |
|  |  | Percentage Correct | 100% |
|  |  | Score | 1 |
|  | | | |
| 13 | *Is there a well-functioning communication system between distribution points?* | Confirmation of effective communication system by all points in the distribution | 1 |
|  |  | Percentage Correct | 100% |
|  |  | Score | 1 |
