# Supplementary material for: Transparency in Nigeria's public pharmaceutical sector: perceptions from policy makers
Source: Global Health. 2009 Oct 29;5:14. doi: 10.1186/1744-8603-5-14 (PMC2775729; doi:10.1186/1744-8603-5-14)
Supplement: Additional file 3 — Organogram of the subdivisions of NAFDAC. [file 1744-8603-5-14-S3.docx]

# Transparency in Nigeria’s Public Pharmaceutical Sector: Perceptions from Policy Makers

*Habibat A Garuba, Jillian C Kohler, Anna M Huisman*

**Additional File 3**

**Title:** Organization structure of the National Agency for Food and Drug Administration and Control (NAFDAC)

**Description:** The flow chart below depicts the organizational breakdown of the directorates and functional units of the NAFDAC

**National Agency for Food and Drug Administration & Control**

**(NAFDAC)**

**Office of the Director-General**

**Directorates**

Finance and Accounts

Planning, Research, and Statistics

Registration and Regulatory Affairs

Narcotics and Controlled Substances

Ports Inspectorate

Establishment Inspectorate

Laboratory Services

Enforcement

Administration and Human Resources

**Functional Units**

Food and Drug Information Centre (FDIC) and Pharmacovigilance (newly created)

Technical Services

Public Relations

Legal Services

Internal Audit

Director General Lagos Liaison Office
